# New Method Based on Direct Analysis in Real-Time Coupled with Time-of-Flight Mass Spectrometry (DART-ToF-MS) for Investigation of the Miscibility of Polymer Blends

**DOI:** 10.3390/polym14091644

**Published:** 2022-04-19

**Authors:** Mohammed Mousa AlShehri, Zeid A. ALOthman, Ahmed Yassine Bedjah Hadj Ahmed, Taieb Aouak

**Affiliations:** Department of Chemistry, College of Science, King Saud University, Riyadh 11451, Saudi Arabia; mr.researcher2018@gmail.com (M.M.A.); zaothman@ksu.edu.sa (Z.A.A.); ybadjah@ksu.edu.sa (A.Y.B.H.A.)

**Keywords:** miscibility, direct analysis in real-time coupled with time-of-flight mass spectrometry, blends, differential scanning calorimetry, thermogravimetry analysis

## Abstract

The miscibility of a series of binary blends such as polystyrene/poly(methyl methacrylate) (PS/PMMA), polystyrene/poly(vinyl chloride)(PS/PVC), poly(vinyl chloride)/poly(polymethyl methacrylate)(PVC/PMMA) and poly(ethylene-co-vinyl alcohol)/poly(lactide-co-glycolide acid) PEVAL/PLGA with equal ratios and poly(ethylene oxide)/poly(hydroxyl propyl methyl cellulose) (PEO/PHPMC) containing 30 and 70 wt% PEO, which were randomly chosen among the widely systems reported in the literature, was investigated by a new method based on a direct analysis in real-time coupled with time-of-flight mass spectrometry (DART-ToF-MS). To reach this goal these pairs of polymers and copolymers were prepared by solvent casting method. As a first step, the DSC technique was undertaken in this work to highlight the published results on the miscibility of these binary systems. The thermogravimetry analysis (TGA) was used to define the optimum decomposition temperature of these blends programmed for the study of miscibility using the DART-ToF-MS technique. The results obtained by this method based on the comparison of the nature of the fragments resulting from the isothermal decomposition of the blend with those of their pure components have been very effective in demonstrating the character of miscibility of these systems. Indeed, it was found that the PS/PMMA-50 and PS/PVC-50 blends were immiscible, PVC/PMMA-50 and PEVAL/PLGA-50 miscible, and the PEO/PHMC partially miscible. This method, which is rapid and uses a very small amount of sample (1–2 mg) can be extended in its application to other blends whose other methods used have shown their limits due to the intrinsic properties of the polymers involved.

## 1. Introduction

Polymer blending is one of the most appropriate contemporary means for the development of new low-cost polymeric materials which in most cases cannot be obtained by copolymerization. Because the use of this last technique often does not allow control of the composition of the monomeric units in the resulted copolymer and therefore does not allow the desired material to be obtained. In addition, to this, a higher cost is necessary to obtain such material. Adding to this is the higher cost required to obtain such material. However, the manifestation of superior properties depends on the miscibility of homopolymers at the molecular level. Unfortunately, most polymer blends are found to be immiscible. The character of the miscibility of a polymer blend is essential in the choice of a polymer material having new or hybrid properties. It should be noted that a mixture of immiscible polymers results in a fragile material having uncontrolled and undesirable properties. Different techniques are currently used to characterize the miscibility of pairs of polymers in which the most used are the differential scanning calorimetry (DSC) [1,2,3], Fourier transform infrared (FTIR) [4,5,6], viscosimetry [6,7,8], ultrasonic velocity [8] and refractive index [8]. These methods are more or less effective in the miscibility characterization depending on the nature of the polymers involved in the blend. For example, the DSC technique has its limits when the difference between the glass transitions temperatures of polymers involved in the blend is less than 20 °C, the FTIR method loses its effectiveness when the polymers involved in the blend do not have functional groups capable of forming specific interactions that can be detected by this technique. The viscometry technique is not effective when the polymers involved in the blend have identical or close intrinsic viscosities.

So in this case, there is no universal method capable of being used to highlight the miscibility of pairs of polymers. In some cases, the results obtained for the same polymer blends are contradictory in that some authors prove miscibility, others immiscibility, and others indicate partial miscibility. Indeed, the miscibility of poly(vinyl chloride)/poly(methyl methacrylate) (PVC/PMMA) blend was first investigated by Shurer et al. [9]. These authors revealed that such a blend containing PMMA of isotactic and syndiotactic microstructures is partially miscible. On the other hand, this same blend containing atactic PMMA was found perfectly miscible. Other researchers using the DSC method demonstrated that this system was miscible only when the PMMA in the blend was above 60 wt% [10,11,12,13]. The miscibility of polystyrene/poly(methyl methacrylate) (PS/PMMA) blend was studied by different authors. Khan et al. [14] using the viscosimetry and FTIR methods demonstrated that this blend is immiscible regardless of the composition studied. Asser et al. [15] using the DSC technique showed through the presence of two distinct glass transition temperatures for each blend that this blend is immiscible regardless of the composition of this system.

The immiscibility character of the blend involving PVC and PS was proved on the basis of damping [16] and viscosity measurements [17]. The viscosimetry method was proven to be a simple and effective tool for investigating the interactions in dilute solutions of polymer blends [18]. The miscibility of this system was also investigated by different authors [16,19,20,21,22] using different methods such as the DSC, FTIR, viscosimetry, refractive index (RI), and electron microprobe (EMP) analyses. These techniques revealed that the PVC/PS blend was compatible only when the PS/PVC ratio was less than 5/10 wt%. The miscibility of the blend involving poly (ethylene oxide) and poly(hydroxypropyl cellulose)(PEO/PHPMC) was investigated by viscosimetry, ultrasonic velocity, density, and refractive index methods [23,24]. The main result obtained was miscibility when the PEO content in the blend was more than 50 wt%.

In this work, the miscibility of a series of binary blends such as PS/PMMA, PS/PVC, PVC/PMMA, poly(ethylene-co-vinyl alcohol)/poly(lactide-co-glycolide acid) PEVAL/PLGA, and PEO/PHPMC, arbitrarily chosen among the systems widely reported in the literature, was studied by means of a new method based on the direct real-time analysis coupled with the time-of-flight mass spectrometry (DART-ToF-MS). The first two blend systems were chosen according to the literature as immiscible blends, the third as miscible blends, and the fourth as a partially miscible blends. This method is based on the analysis of the fragments resulting from the isothermal decomposition of these blends. To achieve this goal, the miscibility or immiscibility of these blends was initially undertaken by the DSC technique in order to confirm or invalidate their miscibility, then to study the thermal decomposition of these systems by the thermogravimetry method (TGA) in order to identify the optimum temperature of the thermal decomposition of each mixture which will be programmed in the DART-Tof-MS software.

## 2. Experimental Section

### 2.1. Materials

PS ($\bar{M}w\;$= 1.92 × 10^5^ g mol^−1^), PVC ($\bar{M}w$ = 2.33 × 10^5^ g mol^−1^), PMMA ($\bar{M}w$ = 1.20 × 10^5^ g mol^−1^), PVC ($\bar{M}w$ = 2.33 × 10^5^ g mol^−1^), PEVAL ($\bar{M}w$ = 1.0 × 10^5^ g mol^−1^; 32 mol% ethylene), poly(D,L-lactide-co-glycolide acid) (PLGA) ($\;\bar{M}w$ = 3.0 × 10^4^–6.0 × 10^4^ g mol^−1^; lactide:glycolide 50:50), poly(ethylene oxide) (PEO) ($\bar{M}w$ = 6.0 × 10^3^ g mol^−1^) and poly(hydroxylpropyl methyl cellulose) PHPMC ($\bar{M}w$ = 8.60 × 10^4^ g mol^−1^) were provided by Sigma Aldrich (Taufkirchen, Germany). Tetrahydrofuran (THF) (purity, 99%), dimethyl formamide (DMF) (purity, 99.8%), and heptanes (purity, 97) were purchased from AnalaR NORMAPUR (Paris, France). Polymers were purified three times by dissolution in THF and precipitation in heptane, while solvents and precipitants were used without prior purification.

### 2.2. Blends Preparation

PS/PMMA-50, PS/PVC-50, and PVC/PMMA-50 blends containing equal amounts of each polymer and PEO/HPMC blend containing 30 and 70 wt% PEO were prepared by solvent casting method. A known amount of each polymer was dissolved by stirring in 10% by weight THF until complete dissolution. The resulting solution is then filtered on a Waterman filter in order to remove all traces of microgels formed as by-products during the free radical polymerization of the monomer at the origin (the factory). The blends were prepared by mixing the solutions two by two taking into account the composition of the blend targeted by this study. Each ternary system containing two polymers and solvent was poured into Teflon plate molds and left to dry at room temperature (~25 °C) then under vacuum at 50 °C until constant mass. The resulted films of thickness ranging between 235 and 264 µm were placed under vacuum in a desiccator before analysis.

PEVAL and PLGA copolymers were separately dissolved in DMF at 10% by volume to prepare two binary polymeric solutions. Noting that the dissolution of PEVAL in DMF requires heating at 70 °C for 4 h for complete dissolution. PEVAL/PLGA-50 blend containing equal amounts of the copolymer was prepared by mixing together the two polymeric solutions to obtain a PEVAL/PLGA/DMF ternary solution. Copolymers and blend films were prepared by smoothly casting these corresponding polymeric solutions over horizontal Teflon-plate surfaces. To extract the residual solvent clustered in the two copolymers and their blend, the plates were heated at 60 °C for 3 days and then dried under a vacuum at 70 °C for one week. The average thickness of each film measured by a Mitutoyo 293-340-30 Digital Micrometer varied between 50 and 80 μm.

### 2.3. Characterization

#### 2.3.1. DSC

The glass transition temperature of pure components and blends was performed on Shimatzu DSC 60A, previously calibrated with indium. Samples weighing between 10 and 12 mg were packed in aluminum DSC pans before placing them in the DSC cell. The samples were heated from −30 to 240 °C at a heating rate of 10 °C min^−1^. For uniform thermal history in all samples, the thermograms have been represented by the data of the second run, after quenching just above the *Tg*. No degradation phenomena of polymers, copolymers, and their blends were observed in all thermograms obtained. This finding was also confirmed by a test of solubility realized after DSC analysis. The glass transition temperatures were taken at the midpoint in the heat capacity change with temperature and the melting points at the summits of the endothermic peaks.

#### 2.3.2. TGA

Thermogravimetry analysis (TGA) of pure polymers, pure copolymers, and their blends were performed on a TGA/DSC1 Mettler-Toledo thermogravimeter (Columbus, OH, USA). apparatus in a nitrogen atmosphere. Samples weighing between 8 and 14.20 mg were placed in aluminum TGA pans before placing in the TGA cell then scanned in the temperature range 25–600 °C at a heating rate of 10 °C min^−1^.

#### 2.3.3. DART-ToF-MS

The fragments resulting from the isothermal decomposition of blends and their pure components were analyzed by an Accu-ToF LC-plus JMS-T100 LP mass spectrometer JEOL (Tokyo, Japan) working with a direct-analysis-in-real-time (DART) ion source (Ion Sense, Saugus, MA, USA). The samples were used without any prior preparation. The fragments resulting from the decomposition process were evaporated in a stream of helium atmosphere heated at a constant temperature of 350 °C. The heated helium/Vapor mixture was then ionized by excited metastable helium atoms, before entering the ion source of the time-of-flight mass spectrometer. All the samples were analyzed by DART ToF-MS using Accu-TOF mass spectrometer acquired from JEOL (Tokyo, Japan). The experimental conditions were: vacuum level 1.3 × 10^5^ Pa, He used as a heating and ionization gas, ring lens with a voltage of 4 V, peaks voltage of 500 V, and the mass resolution was ranged between 3600 and 4900. The sample (1–2 mg) in powder form was placed in a small piece of sandpaper and introduced between the DART outlet and the MS port inlet.

## 3. Results and Discussion

### 3.1. DSC Analysis

The study of the miscibility of PS/PMMA-50, PS/PVC-50, PVC/PMMA-50, and PEVAL/PLGA-50 blends containing equal ratios in polymer and PEO/PHPMC with 30 and 70 wt% PEO was studied briefly by the DSC method. The demonstration of the miscibility of a polymer blend by this technique is based on the appearance of an ultimate Tg on its thermogram between those of their pure components, while its immiscibility is evidenced by the appearance of two Tgs belonging to the pure components. The purpose of this study by DSC method in this work is to follow up with the studies already invested by different authors on the miscibility of these systems using different methods and also to confirm or refute the results obtained before starting the main subject of this study, which is the use of the DART-Tof-MS technique as a new way to study the miscibility of polymer blends.

(A) Miscibility of PS/PMMA-50

**Figure 1:**
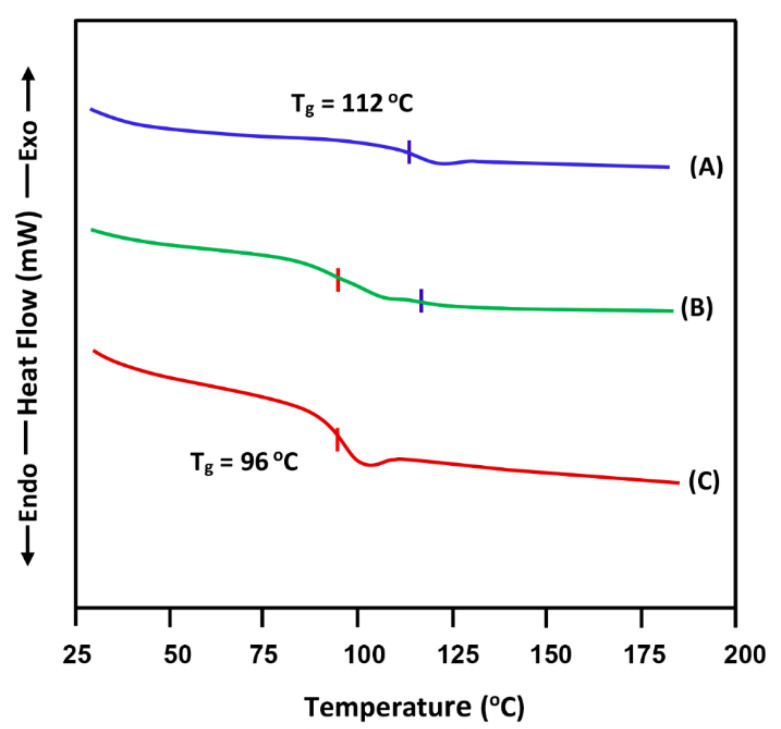
DSC thermograms of: (A) pure PMMA; (B) PS/PMMA-50 blend and (C) pure PS.

Analysis of the miscibility of the PS/PMMA-50 blend by the DSC method led to the results of the thermograms in Figure 1. As can be seen through these thermal curves, the pure PMMA and PS components show Tgs at 112 °C and 96 °C, respectively, which are in agreement with the literature [25,26]. On the other hand, the thermogram of the blend reveals an immiscible character by the presence of two Tg specific to pure polymers (Table 1), thus confirming the results obtained by Chuai et al. [27] and Khan et al. [14] using the viscosimetry method.

(B) Miscibility of PS/PVC-50

**Figure 2:**
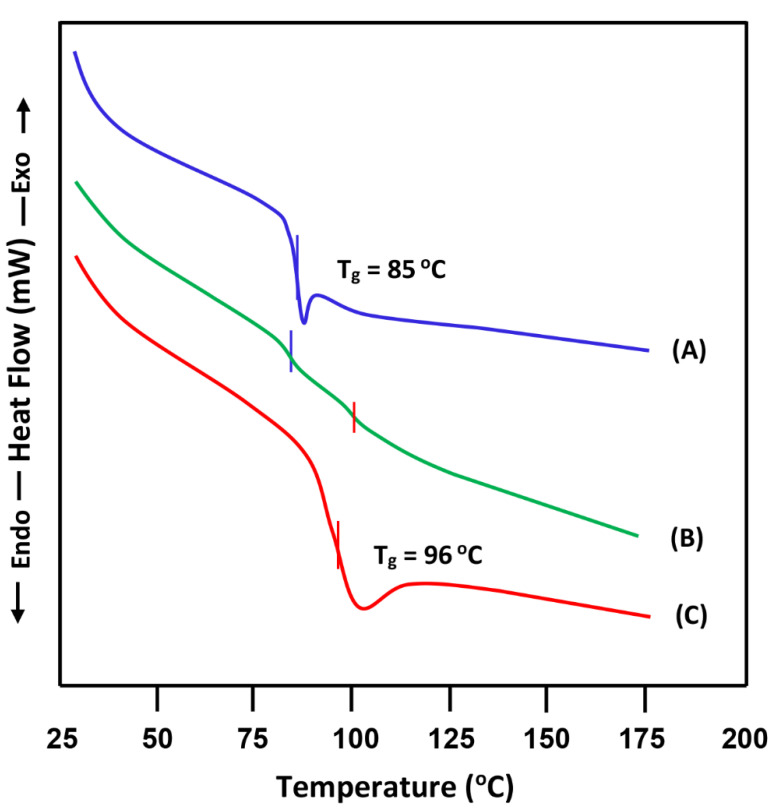
DSC thermograms of: (A) pure PVC; (B) PVC/PS-50 and (C) pure PS.

Regarding the PS/PVC-50 blend, the DSC thermogram of this system as shown in Figure 2, and the Tgs values deducted are summarized in Table 1. The profile of the thermal curve of the blend reveals two Tgs belonging to the pure polymers, indicating its immiscible character in this composition. This confirms the results obtained by Zhonk et al. [28] using the same method, and Pinping et al. [16] using the viscosimetry technique.

(C) Miscibility of PVC/PMMA-50

**Figure 3:**
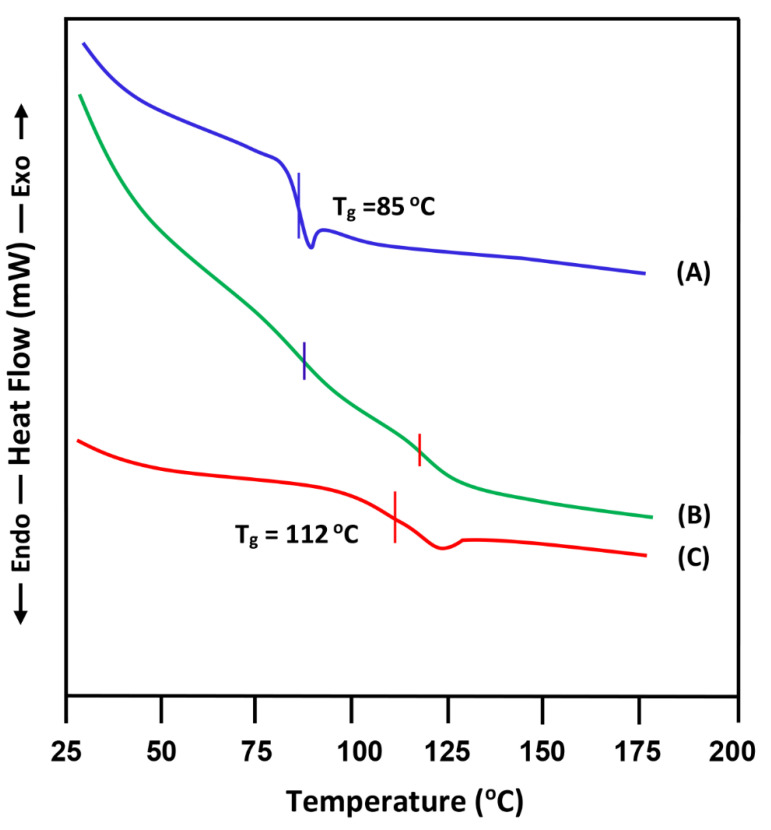
DSC thermograms of: (A) PVC; (B) PVC/PMMA-50 and (C) PMMA their pure components PVC and PMMA.

The DSC thermograms of PVC/PMMA blend with equal polymer ratio are gathered with its pure components in Figure 3 and the Tg values obtained are grouped in Table 1. As shown through the comparison of these thermal curves, this blend shows in its thermogram two Tgs which coincide with those of their pure components, indicating the immiscible character of this system in this composition. According to the literature, the miscibility of the PVC/PMMA system is controversial. For example, Deshpande et al. [5] have shown by thermal studies that the blend cast from methylethylketone was immiscible for 50 wt% of PVC content. Belhaneche-Bensemra et al. [10] investigated the miscibility of this system by DSC and FTIR and reported that the miscibility of this system is miscible when the PMMA content in the blend was more than 60 wt%. On the other hand, Khan et al. [14], using the viscosimetry and FTIR methods, suggest the miscibility of this system over the whole composition range. The investigations concerning the PVC/PMMA blend reported by others authors have demonstrated that this system became immiscible beyond 70 wt% of PMMA [9,29,30,31]. The results obtained in this work reflect well those of Deshpande et al. [5] in which the immiscibility of this system is observed at 50% by weight of each polymer.

(D) Miscibility of PEVAL/PLGA-50

**Figure 4:**
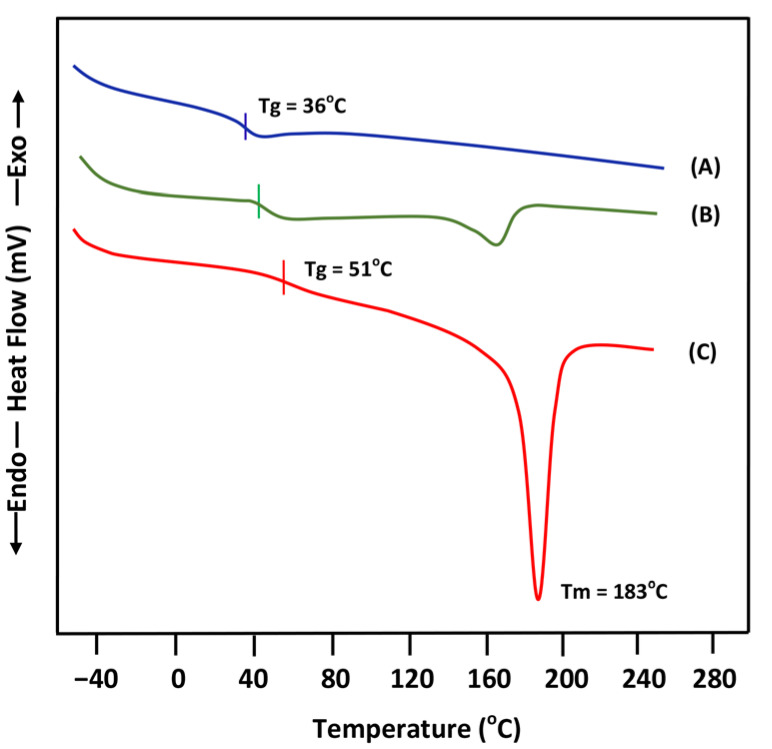
DSC thermograms of pure PLGA(A), PEVAL/PLGA-50 (B) blend and PEVAL (C).

The DSC thermograms of the blend involving 50 wt% of PEVAL and an equivalent amount of PLGA are presented with their pure components in Figure 4 and the Tg values collected are shown in Table 1. The curve profile of the blend shows a confusing zone between 30 and 62 °C, in which the Tg value cannot be precisely located, due to the small difference that exists between the Tgs of their pure components, PEVAL (51 °C) and PLGA (36 °C), which does not exceed 20 °C. Therefore, despite the presence of a shift in the Tg value, this small difference cannot be rigorously used as proof of miscibility using the criterion based on the unique Tg of the blend. This same thermogram also reveals an important depression in the melting temperature of PEVAL in the blend from 183 to 174 °C when 50 wt% of PLGA was added. This is probably due to a decrease in the crystallinity of the PEVAL in the mixture when a significant part of the PLGA chains has infiltrated between the chains of the copolymer. This is favored by the formation of hydrogen bonds between the carbonyl oxygen belonging to the PLGA and the hydroxyl of the PEVAL. During our bibliographical search, we did not find any studies using other techniques on the miscibility of this system to support this observation, except that carried out by our team [32] using this same method and that of FTIR revealing the presence of hydrogen bonds between the hydroxyl of PEVAL and the carbonyl of PLGA.

(E) Miscibility of PEO/PHPMC

**Figure 5:**
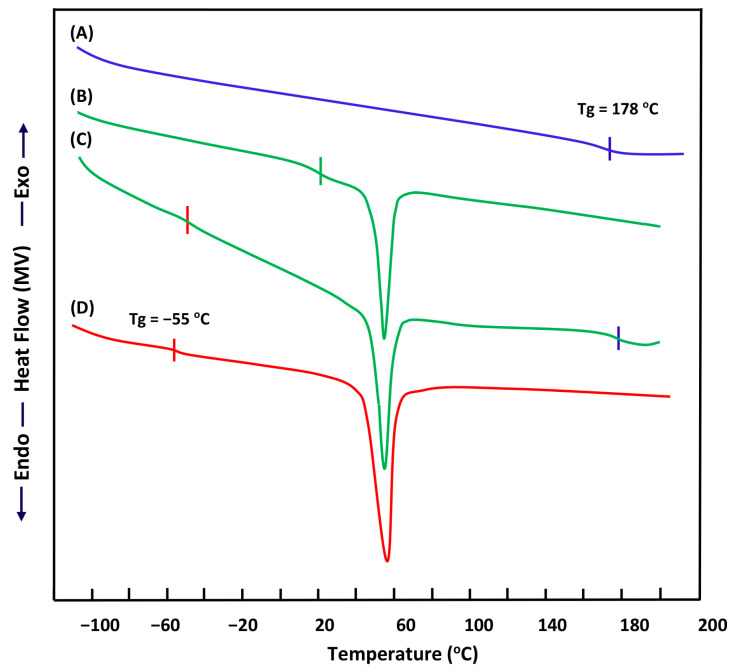
DSC thermograms of: (A) pure PHPMC; (B) PEO/PHPMC-70; (C) PEO/PHPMC-30 and (D) pure PEO.

The study of the miscibility of the PEO/PHPMC system containing 30 and 70 wt% PEO by the DSC method led to the thermograms of Figure 5 and the Tgs values are deducted are summarized in Table 1. These thermal curves show the pure PEO and pure PHPMC Tg values at 65 °C and 178 °C, respectively, which both agree with the literature [33,34]. On the other hand, the thermogram of the blend containing 30 wt% PEO shows two distinct Tgs specific to the pure components, thus indicating the immiscible character of this system in this composition, while, that with 70 wt% PEO shows an ultimate Tg between those of its pure components thus proving the miscibility of this system. Such behavior was also observed by Basavaraju et al. [23] using the viscosimetry, ultrasonic, refractive index, and density measurements. These authors concluded that this system remains miscible when PEO incorporated in this blend was greater than 50 wt%. On another side, Fuller et al. [35] demonstrated the miscibility of this system in all proportions by means of DSC through the negative value of the Flory–Huggins interaction parameter, χ, determined from the Nishi and Wang equation [36]. These authors have also confirmed the presence of a hydrogen bond between the two polymers by FTIR which is the origin of the miscibility.

### 3.2. TGA

**Figure 6:**
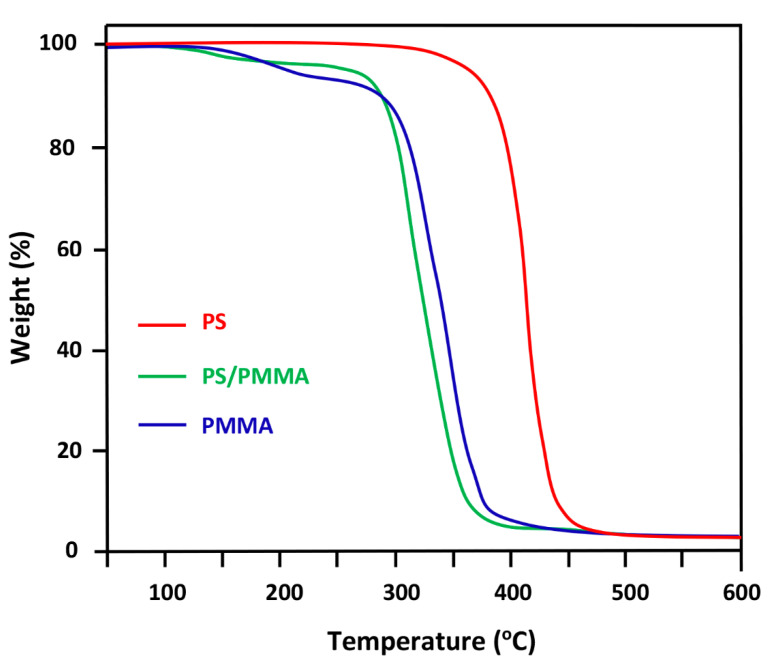
TGA thermograms of pure PS, pure PMMA, and their blend containing 50 wt% of each component.

Figure 6 groups the TGA thermograms of pure PS, pure PMMA, and their blend containing 50 wt% of each component. These thermal curves show only one decomposition temperature for all samples and the pure PS appears to be the most thermally stable. The onset of thermal decomposition of PS is observed at 240 °C, while that of PMMA is at 220 °C. The thermal stability of the equal ratio PS/PMMA blend retrogrades to reach a minimum at 205 °C. Similar results were also obtained by Kaniappan et al. [37] on the thermal stability of this system.

**Figure 7:**
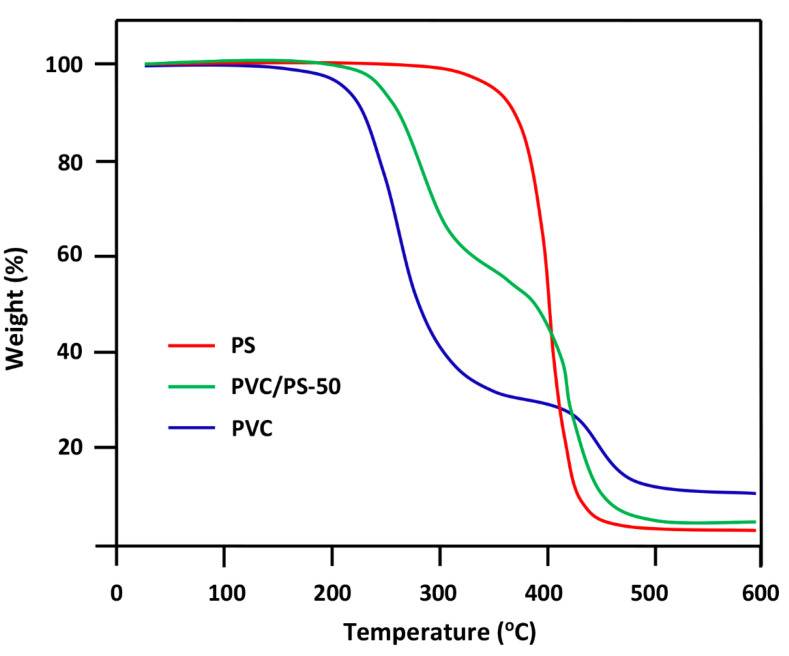
TGA thermograms of PVC/PS-50 blend and their pure components PVC and PS.

The TGA thermogram of PS/PVC blend with 50 wt% by of each component and those of its pure components are gathered for comparison in Figure 7. As it can be seen from these thermal curve profiles the degradation process of pure PVC passed through two main steps. The first one started at about 200 °C for the first decomposition and at 435 °C for the second. This result was also observed by different authors [38,39,40] and attributed the decomposition of this polymer between 220–400 °C to the elimination of HCl molecules and between 400 and 475 °C to the scission of principal chains leading to lower molecular weight fragments. However, PS, which is more thermally stable, degrades in a single step in a continuous process that starts at 425 °C. During this step, polystyrene decomposes to styrene monomer, dimer, and trimer [41]. According to these same authors, slight retardation of HCl release from PVC occurred when it was blended with 50 wt% of PS. This is due to the intermolecular transfer of Cl radicals to both PVC and PS thus, the Cl radicals which initiate the dehydrochlorinations decreased [33,42].

**Figure 8:**
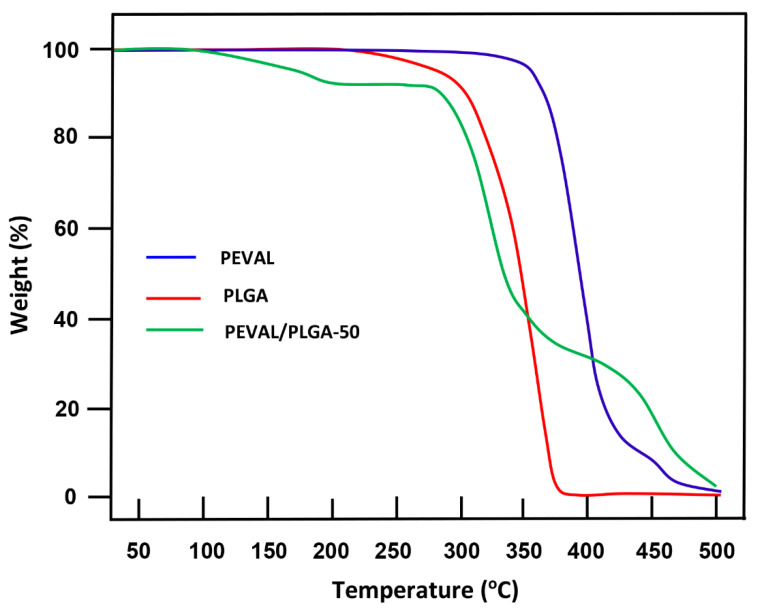
Thermograms TGA of pure PEVAL, PLGA, and their blend with equal ratio.

Figure 8 shows the TGA thermal curves of pure PEVAL, pure PLGA, and their blend containing an equal amount of each copolymer. The thermal curve of the mixture reveals that the decomposition of this system goes through two distinct stages. The first stage starts at 270 °C and the second at 350 °C. The weight drop observed at 150 °C is probably attributed to the evaporation of free water incrusted in the blend. Alvarez et al. [43] revealed that the thermal decomposition of PEVAL observed at temperatures ranging between 350 and 412 °C, is mainly due to the decomposition of the alcoholic sequences of this copolymer. Holland et al. [44], reported that the products resulting from the thermal decomposition of poly(vinyl alcohol)(PVA), in this temperature range, chain scission through a six-membered transition state occurred leading mainly to the formation of the volatile products; such as aldehydes and ketones. When the temperature was superior to 412 °C, a decomposition of co-component polyethylene occurred, leading to a release of ethylene and other volatile hydrocarbon compounds. Sivalingam et al. [45], demonstrated that the PLGA exhibited a decomposition mechanism that could involve random chain scissions at the beginning of the degradation and specific chain scissions at the end. At temperatures below 412 °C, the decomposition of PLGA follows the main degradation mechanism which is inspired by those of PLG and PLA involving transposition reactions of non-radical esters of the backbiting type incorporating the hydroxyl in the end of the chain. This process leads mainly to the formation of linear and cyclic oligomers, regeneration of monomers, and other products such as acetaldehyde, methylglycolate. Carbon dioxide is also a decomposition product resulting from the decarboxylation reactions of the terminal carboxyl groups of the polymer chains [46].

**Figure 9:**
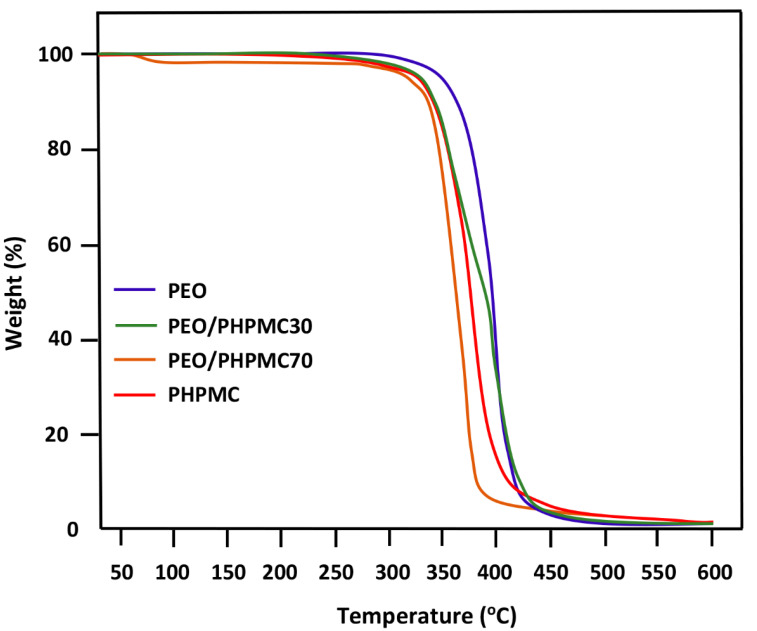
TGA thermograms of PEO/PHPMC blends and their pure components PEO and PHPMC.

The thermal decomposition of PEO/PHPMC blends containing 30 and 70 wt% PEO led to the results in Figure 9. The profiles of the curves attributed to the pure polymers show only a single decomposition step which starts at 370 °C for PEO and 335 °C for PHPMC, indicating that the PEO is slightly more thermally stable than PHPMC. Such a result was also reported in the literature [14]. However, the thermal curve of the blend with 30 wt% PEO shows two stages of decomposition, the first one begins, as in the case of pure PHPMC, at 335 °C and the other at 370 °C. While the profile of the blend with 70 wt% PEO presents only an ultimate stage of decomposition which starts at 303 °C, indicating a loss of 67 and 32 °C in the thermal stability of this blend compared to PEO and PHPMC, respectively, i.e., approximately twice that of the PEO.

### 3.3. DART-ToF-MS

**Scheme 1:**
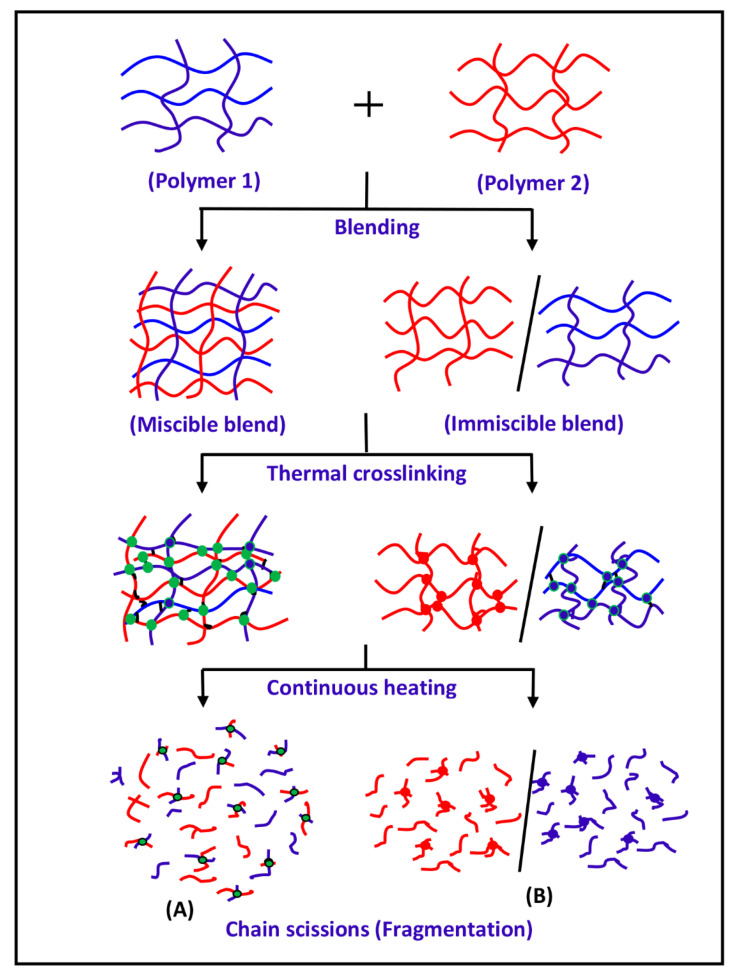
Suggestion of an isothermal decomposition process of (**A**) miscible and (**B**) non miscible polymer blend.

The miscibility study of the PS/PMMA, PS/PVC, PVC/PMMA, PEVAL/PLGA, and PEO/PHPMC blends was carried out by the DART-ToF-MS method based on the comparison of the fragments generated from the decomposition isothermal of the blends with those of their pure components. Assuming that, in a miscible blend, the chains of different natures involved in the blend are distributed uniformly in the polymer matrix, therefore only one phase containing the two components is obtained. As shown in Scheme 1, the thermal degradation of a miscible blend first leads to the formation of a crosslinked network involving the two types of polymer chains according to a radical reaction mechanism. In the second step, the resulted network decomposes according to intrachain and interchain scissions of neighboring chains generating fragments involving the two different polymer chains (Scheme 1A). On the other hand, in an immiscible blend, the chains of different nature repel each other and the chains of the same polymer join together, leading to the formation of two distinct phases, each phase is rich in each polymer. In this situation, the isothermal degradation of such a blend leads to the formation of two distinct crosslinked networks (Scheme 1B). Under these conditions, the decomposition reactions of this immiscible blend only generate the fragments resulting from the intrachain and interchain scissions of the pure components. 

**Figure 10:**
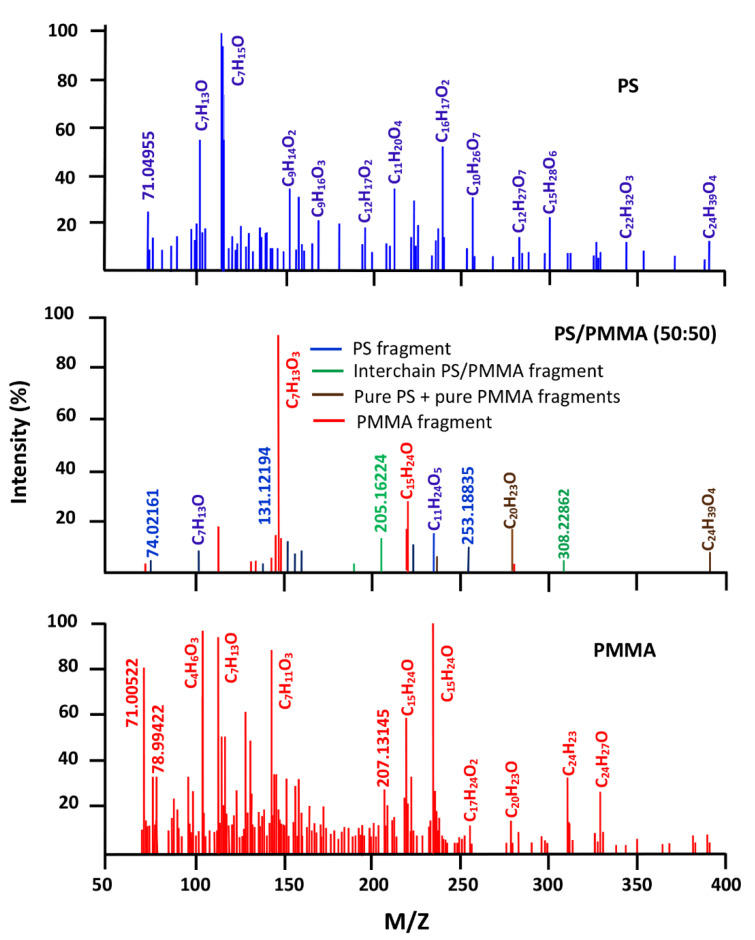
Comparative spectra of pure PS, PMMA, and their blend with 50 wt% of each component.

In this work, the application of the proposed method to highlight the character of miscibility behavior of these binary systems gave the following results:

The isothermal decomposition of the PS/PMMA-50 blend led to the data in Figure 10. As shown from the comparison of the signals attributed to the blend with those of pure components, the signals belonging to these two involved polymers are depicted, and only two small signals indicate the generation of new fragments probably resulting from the interchain scissions in the border between two different phases involving neighboring chains of different nature (PS-PMMA). This reveals that the chains of the same nature are grouped together in the blend forming separated phases rich in each polymer (Scheme 1B). Interchain scissions occur after or during the cross-linking reactions between two or more neighboring chains involving a radical mechanism. This result confirms the immiscible character of PS/PMMA-50 thus confirming the results of the literature obtained by viscosimetry, FTIR, and DSC methods [22,27].

**Figure 11:**
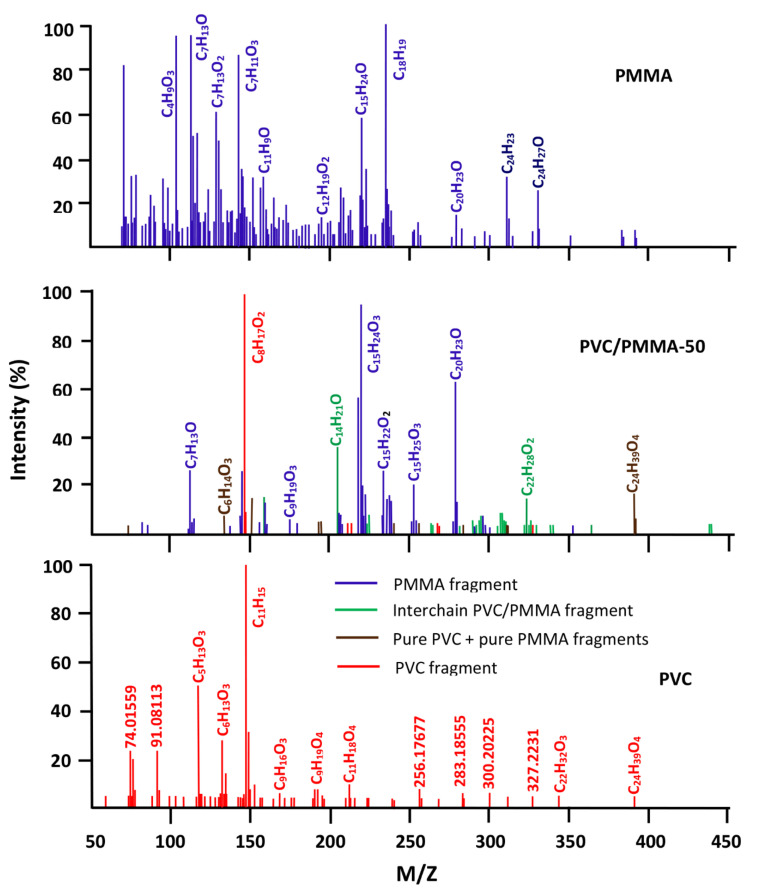
Comparative spectra of pure PVC, PS, and their blend with 50 wt% of each component.

The isothermal decomposition of the PS/PVC-50 blend and those of its pure components gave the results in Figure 11. Comparison of the spectrum of the mixture with those of their pure components reveals the presence of signals attributed to the fragments resulting from the interchain and intrachain scissions of the two pure polymers. Moreover, this spectrum, like the case of the PS/PMMA mixture studied previously, also reveals a small number of low-intensity signals revealing the presence of a small number of segments generated by the scission of the chains of the cross-linked interpenetrating network involving the PVC and the PS present at the interface between the different phases. This proves the immiscible character of this blend, thus confirming the results of the literature obtained by viscometry, RI, and FTIR methods [14,16,47].

**Figure 12:**
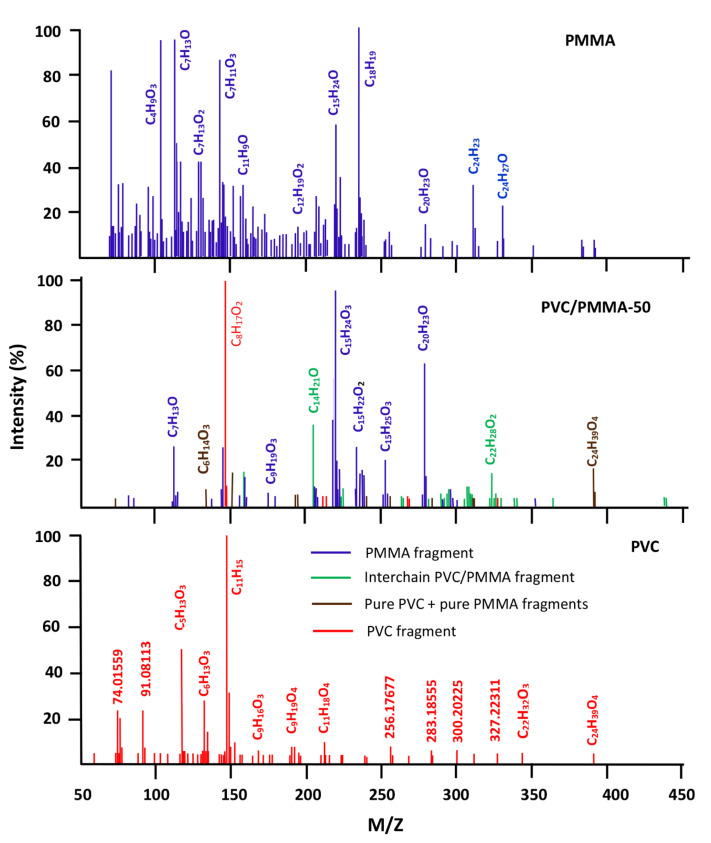
Comparative spectra of pure PVC, PMMA, and their blend with 50 wt% of each component.

The DART-ToF-MS spectra of PVC/PMMA-50 and their components are shown in Figure 12. As it can be seen from these data, the spectrum of the blend shows most of the signals belonging to the fragments resulting from the decomposition of the two pure polymers, as well as others less important in number and intensity attributed to the fragments generated from the scission of the PVC-PMMA chains in the boundary between different phases. This finding highlights the immiscible character of the PVC/PMMA-50 blend as reported by different authors using DSC, FTIR, and viscosimetry methods [10,11,12,13,14,15].

**Figure 13:**
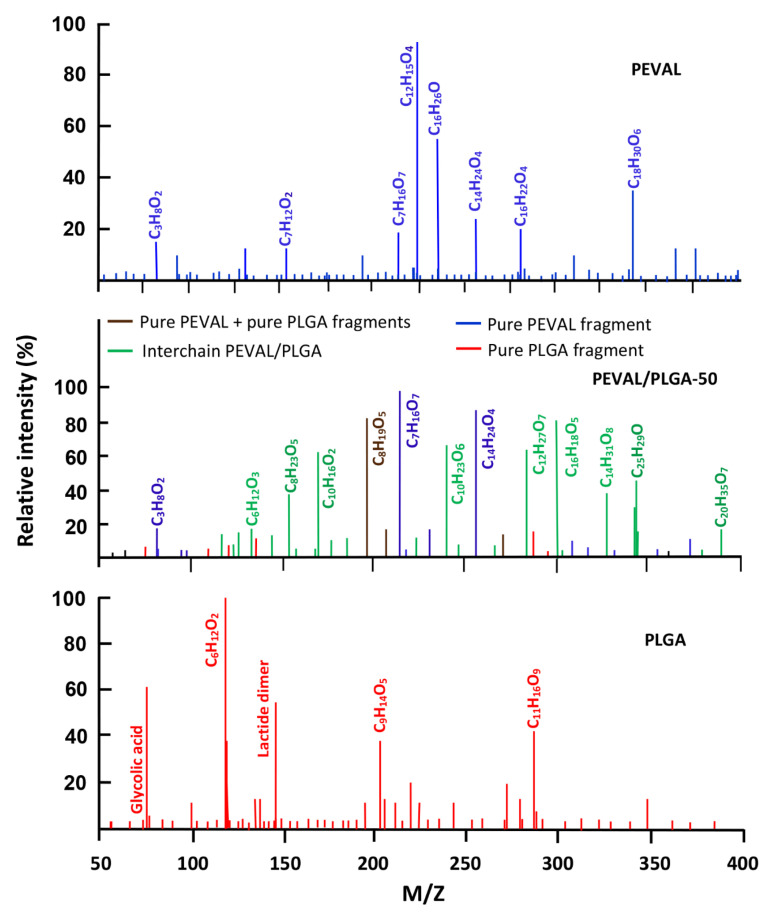
Comparative spectra of pure PEVAL, PLGA, and their blend with 50 wt% of each component.

The spectra obtained from the isothermal decomposition of the PEVAL/PLGA-50 blend and their pure components by DART-Tof-MS analysis are grouped in Figure 13. The comparison of the signals of the blend with those of their pure components reveals, in addition to the signals belonging to the fragments resulting from the intrachain scissions of the two pure components (PEVAL-PEVAL and PLGA-PLGA) in the blend (in blue and in red), other new signals (in green) attributed to fragments resulting from the interchain scissions of the crosslinked network involving chains of different nature. This reveals a homogeneous dispersion of the chains of different nature in the blend, thus demonstrating the miscible character of this pair of polymers in the composition studied. Such behavior has also been demonstrated by FTIR and DSC in a recent article in the literature [47].

**Figure 14:**
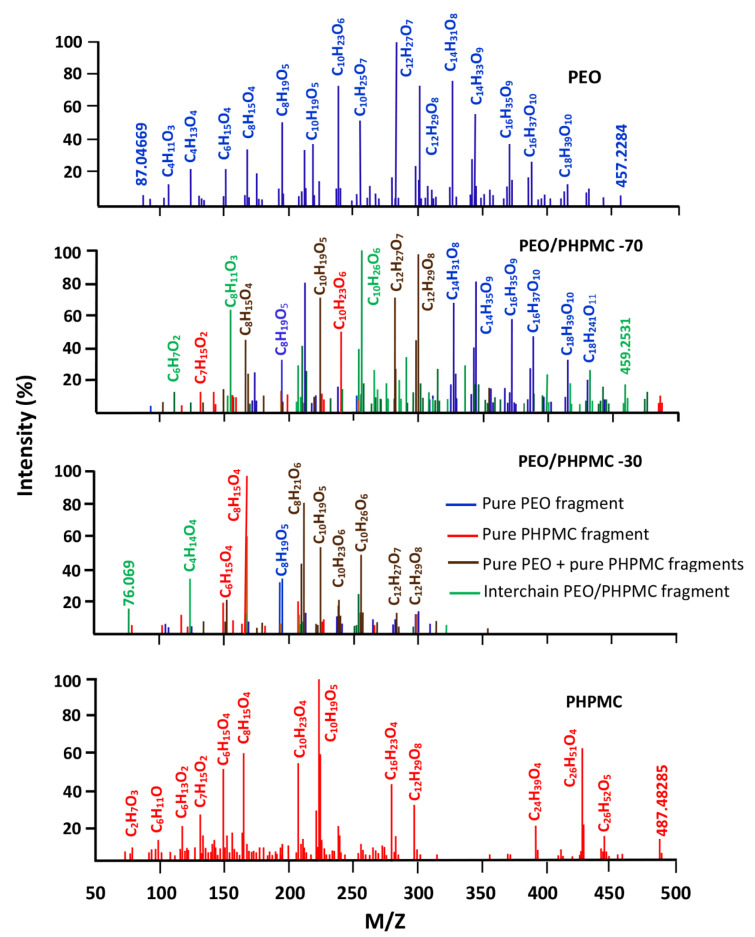
Comparative spectra of pure PEO, PHPMC, and their blend with 30 wt% and 70 wt% of PEO.

The results of the thermal decomposition of the PEO/PHPMC system containing 30 and 70 wt% PEO and those of their pure components obtained by the DART-Tof-MS method are presented in Figure 14. As it can be seen from the spectrum of the blend with 30 wt% PEO, new signals (in green) belonging to new fragments resulting from interchain scissions involving the two interpenetrated network polymers, in addition to those generated from intrachain scissions within the pure polymers (in brown, blue and red). This result reveals the miscible behavior of this blend in this composition. However, the blend containing 70% PEO presents practically only the signals belonging to the two pure polymers (in brown, blue and red) thus revealing the immiscible character of this blend in this composition. Such behavior was also observed by Basavaraju et al. [23] using viscosimetry, ultrasonic, refractive index, and density measurements. These authors concluded that this system remains miscible when PEO incorporated in this blend is greater than 50 wt%.

Finally, the technique of DART-Tof-MS analysis has shown its efficiency in the study of the miscibility of blends and confirms the results obtained by other methods such as the DSC, FTIR, viscosimetry, refractive index, ultrasonic velocity, density, and refractive index. This method can be extended in its application to other blends whose other techniques used have shown their limits due to the intrinsic properties of the polymers involved. For example, the DSC which is widely used in this field shows its limit when the difference in the Tgs of the polymers involved in the blend is less than 20 °C. This technique also makes it possible to give an important idea about the dispersion of chains of different nature involved in the miscible and immiscible blends.

## 4. Conclusions

The miscibility of PS/PMMA, PS/PVC, PVC/PMMA, PEVAL/PLGA blends with equal ratios, and PEO/PHPMC blends containing 30 and 70 wt% PEO, arbitrarily selected from the literature for miscible and immiscible systems prepared by the solvent casting method has been successfully studied by the DART-ToF-MS method. The DSC method undertaken in this work confirmed once again the miscibility behavior of PVC/PMMA-50 and PEVAL/PLGA-50 blends, the immiscible character of PS/PMMA-50 and PS/PVC-50 blends, and the partial miscibility of PEO/PHPMC system. The optimum thermal decomposition temperature determined by TGA was about 350 °C for practically all investigated blends. The results of the miscibility obtained by the DART-ToF-MS method confirmed the character of miscibility of these systems determined in this work by DSC methods and those of the literature. This method, which is rapid and uses a very small amount of sample (1–2 mg) can be extended in its application to other blends whose other methods used have shown their limits due to the non-adaptable intrinsic properties of the polymers involved. This technique also makes it possible to give an idea of the distribution of the chains of different natures in miscible and immiscible blends.

## Figures and Tables

**Table 1:** Glass transition temperatures of PS/PMMA-50, PS/PVC-50, PVC/PMMA-50, PEVAL-PLGA-50, PEO/PHPMC-30, and PEO/PHPMC-70 blends and their pure components.

System	Tg (°C)	System	Tg (°C)
PS	96	PEVAL	51
PMMA	112	PLGA	36
PS/PMMA-50	86–103	PEVAL/PLGA-50	41
PVC	85	PEO	−55
PS/PVC-50	86–103	PHPMC	178
PVC/PMMA-50	84–118	PEO/PHPMC-30	-
-	-	PEO/PHPMC-70	24

## Data Availability

Data that support the findings of this study are included in the article.
